# Technical note: A 3D‐printed phantom for routine accuracy check of Gamma Knife Icon HDMM system

**DOI:** 10.1002/acm2.12339

**Published:** 2018-05-23

**Authors:** Chuan Wu, Marlyn B. Radevic, Jennifer S. Glass, Stan E. Skubic

**Affiliations:** ^1^ Radiation Oncology Sutter Medical Foundation Roseville CA USA; ^2^ Radiation Oncology Sutter Medical Foundation Sacramento CA USA

**Keywords:** Gamma Knife, HDMM, Icon, phantom, QA

## Abstract

**Purpose:**

To report a novel 3D‐printed device (“SH phantom”) that is designed for routine accuracy check of the Gamma Knife Icon High Definition Motion Management (HDMM) system.

**Methods:**

SH phantom was designed using tinkerCAD software and printed on a commercial 3D printer. We evaluated the SH phantom on our Gamma Knife Icon unit regarding its usability and accuracy for routine HDMM QA.

**Results:**

Single‐axis and multiple‐axis measurements validated the SH phantom design and implementation. An HDMM QA accuracy of 0.22 mm or better along single axis was found using SH phantom.

**Conclusions:**

The SH phantom proved to be a quick and simple tool to use to perform the HDMM system QA. The SH phantom was tested successfully and adopted by us as part of monthly QA for the Gamma Knife Icon.

## 1 INTRODUCTION

In early 2016, the first LekSell Gamma Knife Icon unit (Elekta AB, Stockholm, Sweden) in North America was accepted and commissioned for clinical operation at our institution. Gamma Knife Icon offers a new thermoplastic mask‐based frame‐less treatment option, which can be fractionated if desired. In a typical mask treatment, Gamma Knife Icon uses an infrared camera and reflector system called High Definition Motion Management (HDMM) to monitor patient's intra‐fraction motion. Treatment would be paused if the HDMM detected reflector position was drifting outside of a threshold value (1.5 mm by system default) continuously for more than 2 s.^1^, ^2^, ^3^

Gamma Knife Icon HDMM has a very high accuracy of 0.1 mm or better in ideal conditions, and 0.15 mm when including disturbance factors such as couch movement and HDMM mount vibrations.^1^, ^4^, ^5^ Quantitative quality assurance (QA) of HDMM is essential for proper functioning of the system and is required by California Radiological Health Branch.^6^ However, currently there is no convenient HDMM QA phantom or tool available, either from the manufacturer or third party vendors. In this work, we designed, manufactured, and tested a simple physics phantom to address this issue.

## 2 METHOD AND MATERIALS

**Figure 1:**
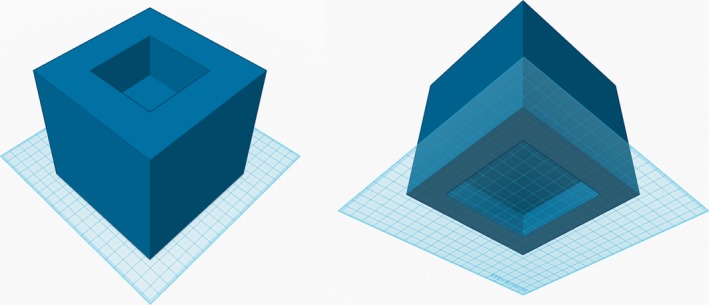
SH phantom base: top view (left) and bottom view (right). Figures are not drawn to scale. The base measures 140 × 140 × 120 (height) mm. The top cutout measures 62 × 62 × 40 (depth) mm. The bottom cutout measures 100 × 100 × 20 (depth) mm.

**Figure 2:**
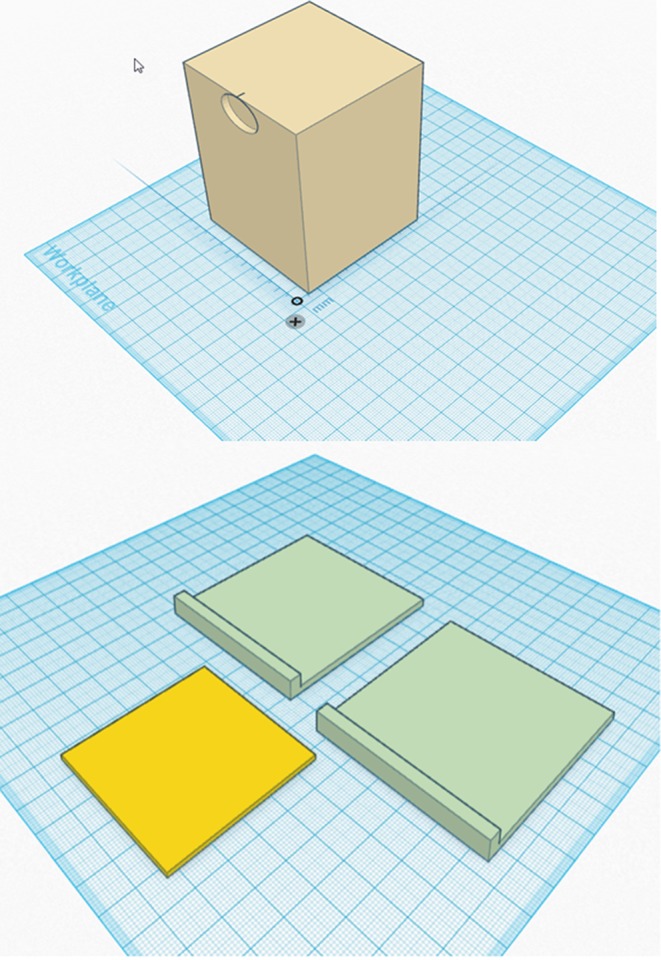
SH phantom reflector stand (top) and thin slice inserts (bottom). Figures are not drawn to scale. The reflector stand measures 60 × 60 × 80 (height) mm. A shallow 2‐mm deep, 20‐mm diameter disk cutout is made on the face facing the HDMM camera. A total of three thin slice inserts were made to create small known shifts for the HDMM QA. Two of them (green in bottom figure) are identical measuring 59 × 65 × 1.7 (thickness) mm, and the other one (yellow in bottom figure) measures 50 × 50 × 1.0 (thickness) mm. The raised edge design on the green inserts aids users to insert or remove them by hand during a QA measurement.

Our phantom is named “SH phantom” (short for Sutter HDMM phantom). It consists of 5 physical parts: the base, the reflector stand, and three thin slice inserts (Figs. 1 and 2).

The SH phantom is designed to be placed on the HDMM head cushion without the thermoplastic mask. Better immobilization would be achieved by having a heavy base, but this would result in an increased material cost. We decided that a base measuring 140 × 140 × 120 mm provided a good compromise.

The top cutout is used to hold the reflector stand (Fig. 2, left). The bottom cutout (instead of a flat bottom) is designed to help immobilize the base of the phantom to the head cushion.

The shallow disk cutout in the reflector stand (top, Fig. 2) helps users properly place a HDMM reflector on the phantom and also to protect it from being damaged during the QA process, when the stand is moved around by hand.

The reflector stand has a cross section of 60 mm × 60 mm, which is 2 mm smaller in both directions than the top cutout in base (62 mm × 62 mm). Theoretically, a 2‐mm thick insert would fit perfectly in this 2‐mm gap — however, due to 3D printer accuracy and material properties, neither the gap nor the thickness of the inserts were exactly 2 mm. To find the best fit, a series of inserts with different thicknesses were printed and tested. The optimal thickness that proved to be reproducible was 1.7 mm.

All 3D design of the phantom was carried out using a CAD freeware (http://www.tinkercad.com). The design was saved in “stl” file format for subsequent 3D printing.

**Figure 3:**
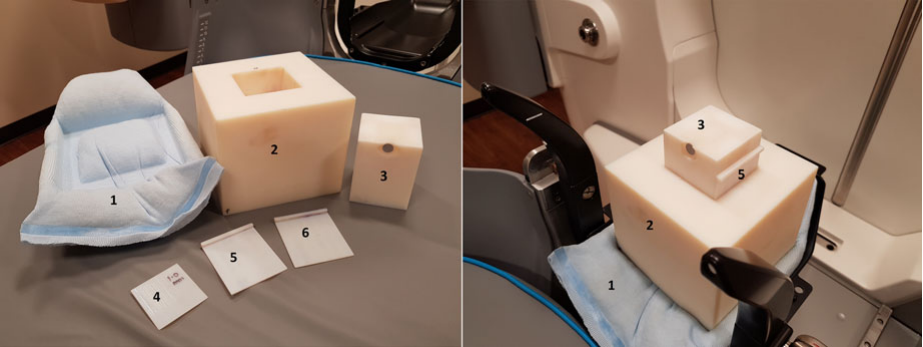
3D‐printed SH phantom parts (left panel) and example QA setup (right panel). The parts are labeled: customized head cushion (1), base (2), reflector stand (3), 1 mm insert (4), two 1.7 mm inserts (5 and 6). In the example QA setup, the 1.7 mm slice (5) is shown inserted to the left side of the reflector stand — this would create a lateral shift to the right as detected by HDMM.

A Polyjet 3D printer (Objet Connex 260 V) was used for printing the SH phantom, using its rigid white proprietary acrylate based polymer material. The printer has an accuracy of 0.05–0.15 mm (S. Lucero, private communication). A photo of the actual SH phantom parts and a sample QA setup is shown in Fig. 3.

A Starrett Electronic Digital Micrometer (No. 732XFL) was used to take measurements on 3D‐printed parts. These measurements constitute the expected shift values in Table 1.

**Table 1:** SH phantom measurement results along single axis. The average value and standard deviation from all users is shown. The expected shift is based on micrometer measurements of the 3D‐printed inserts, respectively. The Δ values listed in the third row are discrepancy between measured and expected shift along each axis

	Left (mm)	Right (mm)	Superior (mm)	Inferior (mm)	Anterior (mm)	Posterior (mm)
Expected shift	1.673	1.673	1.669	1.669	0.999	0.999
Measured shift avg. ± std.	1.57 ± 0.08	1.53 ± 0.05	1.87 ± 0.10	1.89 ± 0.08	0.94 ± 0.02	0.94 ± 0.02
Δ	−0.10	−0.14	0.20	0.22	−0.06	−0.06

A total of four users (physicists) participated in the SH phantom data collection for the proposed QA procedure. In a typical QA session, a user would insert specific thin slice insert(s) to create a shift, record the shift from the HDMM screen before and after the insertion, then subtract the two values to obtain the measured shift. Each data point was taken with five measurements.

## 3 RESULTS AND DISCUSSION

Single‐axis measurement results (Table 1) show that by using the SH phantom, an overall QA accuracy level of 0.2 mm or better can be expected, with the best accuracy in anterior–posterior direction and worst in the superior–inferior direction. The difference between accuracies along different axes might be related to the fact that it can be more difficult to manually and consistently insert a slice and create shifts in the superior–inferior or lateral directions.

Gamma Knife Icon HDMM system reports only the composite shift value on screen, thus alignment of the phantom with respect to the Gamma Knife is not of great importance. When the SH phantom was immobilized using the cushion during QA (Fig. 3), it did not need to be aligned perfectly along the stereotactic axis. For example, in Table 1, the reported shifts along lateral axis means they are approximately aligned in left–right direction.

Some additional composite shift measurements along multiple‐axis (2‐axis and 3‐axis) were also taken to help validate the SH phantom. For such measurements, a user would use a combination of thin slice inserts to create a composite shift in a desired direction, then record the HDMM values after the composite shift made. To compute expected value for a composite shift, it is assumed that its component single‐axis shift (Table 1) is orthogonal and thus simple orthogonal vectors addition can be taken. Overall, an accuracy of 0.32 mm was found for our composite shift measurements, which is less accurate comparing to single‐axis measurement (Table 1). This may be due to the fact that more chance of introducing human setup error when manually making composite shifts.

Inter‐users variation, as well as standard deviation from all users (Table 1) were computed and found to be on the order of 0.2 mm. This indicates that the SH phantom design and implementation is valid to common clinical QA practice.

The SH phantom does meet the regulatory requirements for performing a quantitative check of the HDMM system on a monthly basis, as specified by state of California.^6^

Last but not least, the SH phantom is meant to provide a simple quantitative tool for routine QA of Gamma Knife Icon HDMM system. It is a consistency check, instead of an absolute QA measurement — that is, due to its design, SH phantom *cannot* provide a calibrated measurement with an accuracy of 0.1 mm or better to check HDMM. There has been research effort in developing methods to check and verify the absolute accuracy of HDMM: Winch and Johansson^7^ reported a 0.04 mm agreement between HDMM and mechanical increment using Elekta's ball bearing tool with Vernier scale; Chung et al.^5^ reported similar results on HDMM accuracy using “an independent measuring device with a guaranteed accuracy of 0.01 mm”; Wright et al.^8^ reported a 0.06 mm mean accuracy of HDMM by attaching a special tool that can create a known displacement by “turning of a thumb wheel”. However, all methods mentioned above requires special tools or setup and thus impractical for routine HDMM QA. Any absolute or calibrated shifts would probably require a rigid device mounted to the Gamma Knife unit, which is out of scope for this work. SH phantom does, however, provide a practical, easy to use, tool to a clinical gamma knife physicist to confirm that his/her HDMM is performing in an acceptable manner for patient treatment.

## CONFLICT OF INTEREST

The authors declare no conflict of interest.
